# *CDH1* Germline Variants in a Tunisian Cohort with Hereditary Diffuse Gastric Carcinoma

**DOI:** 10.3390/genes13030400

**Published:** 2022-02-23

**Authors:** Jihenne Ben Aissa-Haj, Maria Kabbage, Houcemeddine Othmen, Patrick Saulnier, Haifa Tounsi Kettiti, Amira Jaballah-Gabteni, Azer Ferah, Mouna Medhioub, Amal Khsiba, Moufida Mahmoudi, Afifa Maaloul, Sonia Ben Nasr, Emna Chelbi, Sonia Abdelhak, M. Samir Boubaker, Mohamed Mousaddak Azzouz, Etienne Rouleau

**Affiliations:** 1 Department of Human and Experimental Pathology, Institut Pasteur de Tunis, Tunis 1002, Tunisia; maria.kabbage@pasteur.utm.tn (M.K.); haifa.tounsi@gmail.com (H.T.K.); amira.jaballah@pasteur.utm.tn (A.J.-G.); jheyna@live.com (A.M.); boubaker.samir@yahoo.fr (M.S.B.); 2 Laboratory of Biomedical Genomics and Oncogenetics, Institut Pasteur de Tunis, University of Tunis El Manar, Tunis 1002, Tunisia; sonia.abdelhak@pasteur.utm.tn; 3 Sydney Brenner Institute for Molecular Bioscience, University of the Witwatersrand, Johannesburg 2000, South Africa; houcemoo@gmail.com; 4 Genomic Platform Molecular Biopathology Unit, URA3655 Inserm, US23 CNRS, Gustave Roussy, 94805 Villejuif, France; patrick.saulnier@gustaveroussy.fr; 5 Laboratory of Venoms and Therapeutic Biomolecules, LR16IPT08 Institut Pasteur de Tunis, University of Tunis El Manar, Tunis 1002, Tunisia; azer.farah@gmail.com; 6 Gastroenterology Department, Mohamed Tahar Maamouri Hospital, Nabeul 8000, Tunisia; medhioub.mouna@yahoo.fr (M.M.); amal.khsiba@yahoo.fr (A.K.); mahmoudi.moufida@gmail.com (M.M.); mm.azzouz@rns.tn (M.M.A.); 7 Faculty of Medicine Tunis, University of Tunis El Manar, Tunis 1068, Tunisia; 8 Oncology Department, Military Hospital of Tunis, Tunis 1008, Tunisia; sonia.bennasr_res@yahoo.fr; 9 Department of Pathology, Mohamed Tahar Maamouri Hospital, Nabeul 8000, Tunisia; emnachelbi1@gmail.com; 10 Department of Biology and Pathology-Cancer Genetics Laboratory-Gustave Roussy, 94805 Villejuif, France; etienne.rouleau@gustaveroussy.fr

**Keywords:** *CDH1*, *CTNNA1*, germline variants, hereditary diffuse gastric cancer, large rearrangements, Tunisian patients

## Abstract

Mutational screening of the *CDH1* gene is a standard treatment for patients who fulfill Hereditary Diffuse Gastric Cancer (HDGC) testing criteria. In this framework, the classification of variants found in this gene is a crucial step for the clinical management of patients at high risk for HDGC. The aim of our study was to identify *CDH1* as well as *CTNNA1* mutational profiles predisposing to HDGC in Tunisia. Thirty-four cases were included for this purpose. We performed Sanger sequencing for the entire coding region of both genes and MLPA (Multiplex Ligation Probe Amplification) assays to investigate large rearrangements of the *CDH1* gene. As a result, three cases, all with the HDGC inclusion criteria (8.82% of the entire cohort), carried pathogenic and likely pathogenic variants of the *CDH1* gene. These variants involve a novel splicing alteration, a missense c.2281G > A detected by Sanger sequencing, and a large rearrangement detected by MLPA. No pathogenic *CTNNA1* variants were found. The large rearrangement is clearly pathogenic, implicating a large deletion of two exons. The novel splicing variant creates a cryptic site. The missense variant is a VUS (Variant with Uncertain Significance). With ACMG (American College of Medical Genetics and Genomics) classification and the evidence available, we thus suggest a revision of its status to likely pathogenic. Further functional studies or cosegregation analysis should be performed to confirm its pathogenicity. In addition, molecular exploration will be needed to understand the etiology of the other *CDH1-* and *CTNNA1*-negative cases fulfilling the HDGC inclusion criteria.

## 1. Introduction

Gastric Carcinoma (GC) is the fifth most common cancer worldwide, with approximately one million new cases registered in 2018 (5.7%) and a wide variation in geographical distribution. It represents the third leading cause of death from cancer worldwide, causing 783,000 deaths in 2018, accounting for 8.2% of all cancer deaths [1,2]. Gastric tumors are histologically and genetically heterogeneous, likely because of the exposure of populations to different environmental risk factors and different genetic predispositions. Despite a decline in incidence and mortality, the burden of GC remains relatively high [3]. Incidence predominates in populations from certain geographic regions and socioeconomic groups [4,5]. High-incidence areas include East Asia, Eastern Europe, Central and South America, Japan, and Korea, while low incidence rates are observed in South Asia, North and East Africa, and North America [6,7].

In Tunisia, GC is the seventh most frequently diagnosed cancer with an incidence of 4% (637 new cases per year), and the fifth most common cause of death with a rate of 5.8% [1,2], and it lacks epidemiological data on hereditary forms.

Histologically, GC is divided into three main subtypes: intestinal, diffuse, and mixed, which have different epidemiological and prognostic features [8,9,10]. Sporadic gastric tumors represent 90%, and familial clustering is rare, representing about 10%. Only 1 to 3% are hereditary [11,12] including several syndromal forms, such as familial intestinal gastric cancer (FIGC) and Hereditary Diffuse Gastric Carcinoma (HDGC) (OMIM: 137215). HDGC is an autosomal dominant inherited disorder caused by germline mutations of the *CDH1* gene with a risk of developing a diffuse type starting at age 45 [13]. *CDH1* mutation carriers have a 70–80% lifetime risk of developing GC [14]. To date, according to the Human Gene Mutation Database (HGMD), more than 155 mutations resulting in loss of function of the *CDH1* gene have been reported worldwide [14,15,16]. However, no hotspots have been characterized.

E-cadherin (OMIM: 192090), a *CDH1* gene product that belongs to the cadherin superfamily, is a calcium-dependent cell–cell adhesion molecule that plays a critical role in the establishment of epithelial architecture, maintenance of cell polarity, and differentiation. It consists of a single transmembrane domain linked to a cytoplasmic domain and an extracellular domain consisting of five tandemly repeated domains called EC1–EC5, which are exclusive to the cadherin family [12,17,18]. According to the International Gastric Cancer Linkage Consortium (IGCLC), patients who meet the inclusion criteria for HDGC must be tested for *CDH1* germline mutations [15]. However, approximately 14–50% of cases meeting the IGCLC inclusion criteria are carriers of pathogenic germline mutations of the *CDH1* gene [14,15,16]. Several families meeting the HDGC inclusion criteria have no detectable pathogenic *CDH1* variant. Other candidate genes, such as *CTNNA1*, have been identified. *CTNNA1* encodes for α-catenin, an E-cadherin partner that plays an important role in the cell adhesion process [19].

To the best of our knowledge, no previous study on HGCs has been performed to identify the mutational spectrum, neither in Tunisia nor in other North African countries. This study set out to identify the genetic mutational profiles of *CDH1* and *CTNNA1* genes in Tunisian patients with DGC to find a new tool for molecular screening of individuals at high risk. To do so, we selected a cohort of 34 cases of DGC with suspected HDGC meeting or not meeting the IGCLC testing criteria.

## 2. Materials and Methods

### 2.1. Study Population

This study was conducted in accordance with the Declaration of Helsinki and with the approval of the Institutional Review Board (IRB) of Institut Pasteur de Tunis. It was a retrospective and consecutive study that included 34 unrelated Tunisian consenting patients between 2009 and 2019. Of these included cases, 22 fulfilled the 2015 international guidelines for *CDH1* genetic screening [14]: (1) two or more GC cases regardless of age, at least one confirmed DGC, in first-degree and second-degree relatives; (2) one case of DGC before 40 years old; (3) personal or familial history of DGC and lobular breast cancer (LBC) with at least one diagnosed before the age of 50 years. Twelve cases did not fulfill the 2015 HDGC clinical testing criteria. Blood samples were collected from 33 index cases and their consenting relatives in the gastroenterology department of Hospital M. Tahar Maamouri-Nabeul and one case in the oncology department of the Military Hospital of Tunis.

### 2.2. Molecular Analysis 

#### 2.2.1. DNA Isolation 

Total genomic DNA (gDNA) was isolated from peripheral blood before any treatment using the salting-out method or the DNeasy^®^ Blood and Tissue Kit from Qiagen according to the manufacturer’s instructions. Somatic DNA (sDNA) was isolated from tumor tissues and was performed using the AllPrep DNA/RNA/Protein Mini Kit from Qiagen according to manufacturer’s instructions (Qiagen GmbH, Hilden, Germany).

#### 2.2.2. Primer Design

Primers covering all coding exons and border regions of *CDH1* and *CTNNA1* genes were designed using Primer Express™ Software version 2.0 and amplified by polymerase chain reaction (PCR). Forward and reverse primers contained the extensions 18F tail (ACCGTTAGTTAGCGATTT) and 18R tail (CGGATAGCAAGCTCGT) at their 5′ end [20]. Tails were used to obtain the same annealing temperature [21].

### 2.3. Genetic Analysis of CDH1 and CTNNA1 Genes

Screening of the coding regions of *CDH1* and *CTNNA1* genes was performed using the primers mentioned in Tables S1 and S2, respectively. Sanger sequencing was used to screen both genes for all enrolled cases. The generated data were analyzed using SeqScape version 3.2 (Thermo Fisher, Multiple Life Technologies Corporation, Carlsbad, CA, USA) and BioEdit Sequence Alignment Editor Version 7.2.5 (http://www.mbio.ncsu.edu/BioEdit/, accessed on 4 February 2022). The variants found in our study were described using the recommendations of the Human Genome Variation Society (HGVS) [22], and interpretations were based on the American College of Medical Genetics and Genomics (ACMG’) guidelines [23,24].

### 2.4. Search for Large Deletions/Duplications of the CDH1 Gene Using Multiplex Ligation-Dependent Probe Amplification (MLPA) Assay

Available material (a total of 28 gDNAs and 10 sDNA) was screened for copy number variations (CNV). This was performed using the SALSA P083-D2 *CDH1* MLPA kit (MRC-Holland) according to the manufacturer’s instructions. MLPA products were run on the ABI Prism 3730 xl Genetic Analyzer (Applied Biosystems Thermo Fisher, CA, USA). Results were analyzed using Coffalyser software, (MRC Holland). A dosage ratio (DR) of 1.0 indicates a normal sequence probe; probes with a DR < 0.7 or >1.3 indicate deletions or duplications, respectively, in the corresponding exons.

### 2.5. In Silico Prediction Tools

The predicted effects of identified variants were evaluated using in silico prediction tools to support functional effect and pathogenicity, such as UMD Predictor (http://umd-predictor.eu/, accessed on 4 February 2022), Sorting Intolerant From Tolerant (SIFT) (http://sift.jcvi.org/, accessed on 4 February 2022), PolyPhen-2 (http://genetics.bwh.harvard.edu/pph2/, accessed on 4 February 2022), Protein Variation Effect Analyzer (PROVEAN) (http://provean.jcvi.org/, accessed on 4 February 2022), Mutation Taster (http://www.mutationtaster.org/, accessed on 4 February 2022), FATHMM (http://fathmm.biocompute.org.uk/, accessed on 4 February 2022) and Varsome (https://varsome.com/, accessed on 4 February 2022). All identified variants were classified on the basis of their pathogenicity. All rare variants were cross-referenced with ClinVar (https://www.ncbi.nlm.nih.gov/clinvar/, accessed on 4 February 2022), Leiden Open Variant Database (LOVD) (https://www.lovd.nl/, accessed on 4 February 2022), and UniProt as well as published reports to prioritize them for processing workup. To predict the change of consensus splice sites, we used SPiCE [25]. It combines in silico predictions from Splice Site Finder-like (SSF-like) and MaxEntScan (MES) (2,3) and uses logistic regression to define two optimal decision thresholds: the optimal sensitivity threshold (ThSe) and the optimal specificity threshold (ThSp), 0.115 and 0.749, respectively.

### 2.6. Molecular Modeling Strategy

The structure of cadherin-1 (E-cadherin) has been partially solved. To date, there are 14 available crystal and cryo-EM structures from the Protein Data Bank (PDB), from which we selected the one containing the mutation. We used MODELLER [26] to generate the structure of the mutant. To investigate the functional effects of the mutation, different protocols were applied. The FlexPepDock method from the ROSETTA package was used to refine peptide–protein complexes. The protocol retains 300 structures of the low and high-resolution stages before calculating the energy score. In addition, we used MODPEP [27] to generate an ensemble of conformations that are likely to bind the target for the wild-type and mutant forms. Within the MODPREP workflow, psipred was applied to assign the secondary structure, whereupon the structure of the peptide was assembled using experimentally collected data. In the final stage, molecular dynamics were applied to refine the structures. The ensemble consisted of 1000 conformations, which were then processed for analysis. Finally, we ran an in silico alanine scanning protocol from ROSETTA [28] to calculate the variation in the binding energy (DDG) between two partners after mutating each residue to alanine. Data from the in silico study were analyzed using the MDTraj python library [29].

### 2.7. Immunohistochemistry

To evaluate the expression of E-cadherin, we performed immunohistochemical staining on formalin-fixed and paraffin-embedded (FFPE) samples of gastric tumor tissues. Immunostaining was performed with a primary mouse monoclonal against E-cadherin (NCL-L-E-Cad, clone 36B5, Novocastra TM, Biopole), recognizing the external Nt domain, according to the manufacturer’s instructions using a Novolink Polymer Detection Systems kit (Leica Biosystems, United States/Biopole, Tunisia).

## 3. Results

### 3.1. Characteristics of the Study Population

All tumors were classified as diffuse carcinomas by two independent pathologists. As shown in Table S3 and Table 1, the *CDH1* and *CTNNA1* genes were sequenced for 34 unrelated Tunisian GC patients. The cohort included 13 (38.24%) males and 21 (61.76%) females with a mean age of 48 years at diagnosis (range 23–82 years). There were two patients with a family history of DGC in the first or second-degree relatives, and 15 patients had DGC at ages of less than 50 years. The majority of the patients (14: 41.18%) had an advanced stage of the disease (T3 and T4) (Table 1). According to family history, some families had other cancers, such as BC (37.5%), CCR (37.55%), and other tumors (25%).

### 3.2. Molecular Analysis of CDH1 Gene

#### 3.2.1. *CDH1* Genetic Testing

A total of 34 Tunisian patients with DGC were selected for *CDH1* germline mutationscreening. In the first step, a total of 27 *CDH1* variants (Table S4) were identified and filtered using the following exclusion criteria: (1) do not consider polymorphisms and synonymous variants and (2) exclude variants reported in Clinvar as Benign or Likely Benign. Of the 27 variants, two were novel (c.765G > A and c.1565 + 3_1565 + 4delinsGT) and 10 were classified as polymorphisms because the minor allele frequency (MAF) in the 1000 Genomes database was greater than 1% (c.48 + 6C > T, c.531 + 10G > C, c.1320 + 45G > C, c.1566-80C > G, c.1712-52G > C, c.1896C > T, c.1937-13T > C, c.2076T > C, c.2164 + 17dupA, c.2439 + 52 G > A and c.2634C > T). Coding *CDH1* variants represented 10 out of 27 variants (37.04%), and according to the ClinVar database, variants were classified as benign or likely benign (62.96%), one was described as a variant of uncertain significance (VUS) (3.7%), and two were novel variants (7.4%).

**Figure 1:**
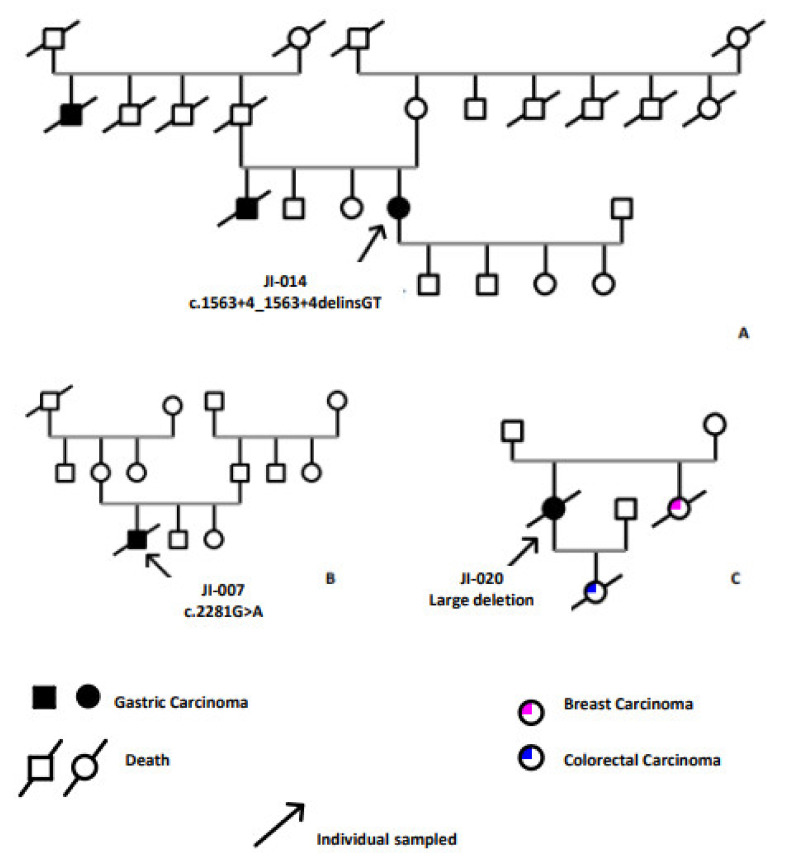
Family history of index cases carrying selected variants. (**A**) Family history of “JI-014” harboring the novel Indel variant c.1563 + 3_1563 + 4delinsGT located in intron 10, predicted to be probably pathogenic. (**B**) Family history of “JI-007” harboring the missense variant c.2281 G > A at exon 14 of the *CDH1* gene, classified as a VUS in the ClinVar database. (**C**) Familial history of “JI-020” carrying the large deletion of two exons (one and two) identified by MLPA assay.

In total, two probably pathogenic variants, c.1565 + 3_1565 + 4delinsGT and c.2281G > A, identified by Sanger sequencing, were predicted to be deleterious by various in silico tools and a pathogenic large deletion, including exons one and two, identified by MLPA assay (Table 2). These three variants were carried out by three different patients meeting the 2015 HDGC clinical testing criteria (3 of 22 patients having clinical testing criteria) (Figure 1).

**Figure 2:**
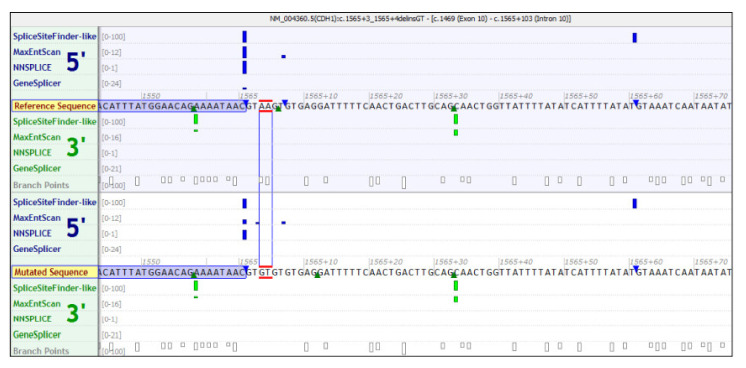
Indel c.1565 + 3_1565 + 4delinsGT effect for the index case JI-014, as shown by Alamut Visual Interactive Biosoftware covering several in silico prediction tools, such as Splice Site Finder-like, MaxEntScan, NNSPLICE, and GeneSplicer.

The first case (JI-014) was a woman who had the novel variant, which is an indel in intron 10 (c.1565 + 3_1565 + 4delinsGT). She was referred for molecular screening for *CDH1*, as she was suspected to have HDGC by the oncology department of the military hospital in Tunisia. She was a 42-year-old woman diagnosed with antro-pyloric DGC (T4N1M1) and treated with palliative chemotherapy. Her brother and paternal uncle were diagnosed with GC and died at the ages of 25 and 80 years, respectively (Figure 1A). She showed a loss of E-cadherin expression. This indel is predicted to affect splice sites. Indeed, the donor site was decreased 3 bps upstream with a percentage of −44.5% (MaxEnt: −64%; NN SPLICE: −25.1%, SSF: −16.8%) resulting in a cryptic site (Figure 2).

**Figure 3:**
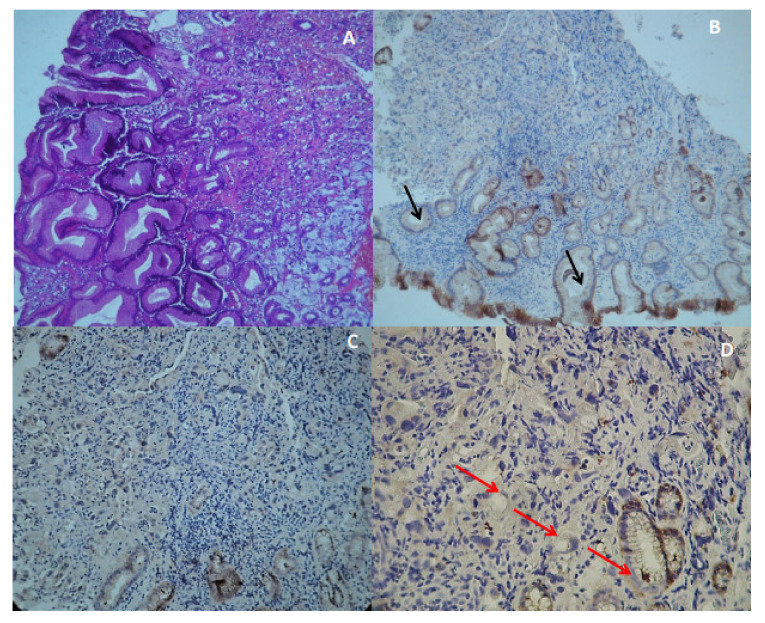
E-cadherin expression status in tumor gastric tissue. (**A**) H and E staining of JI-014 tumor tissue (X100). (**B**) E-cadherin immunostaining expression in gastric tumor tissue (X100). Black arrow shows normal membranous E-cadherin staining in crypt and glandular cells. (**C**) Loss of membranous E-cadherin expression in tumor cells (X200). (**D**) Red arrow shows a loss/reduction of E-cadherin expression in tumor cells and residual glands (X400).

The second index case, JI-007, was a man diagnosed with DGC (T4N0M1) at the age of 25 who died at the same age. He had a silent pedigree (Figure 1B) without a family history of GC or other cancer. This patient carried the predicted probably pathogenic variant (as determined by prediction tools) in the cytoplasmic domain of E-cadherin at exon 14 (c.2281G > A) and showed a loss of E-cadherin expression in gastric tumor tissue (Figure 3C,D). It is a rare variant, rs779648243, with a MAF of 0.0012 in the general population with an uncertain significance in ClinVar. All online prediction tools described the variant as pathogenic.

The third index case, JI-020, carrying the large heterozygous deletion detected by MLPA assay was a 79-year-old woman diagnosed with DGC and treated with total gastrectomy. She had a sister who was diagnosed with BC at age 50 and died at the same age. She also had a daughter who was diagnosed with CCR at the age of 48 (Figure 1C). Because her tumor tissue was unavailable, we were unable to investigate the E-cadherin immunohistological profile.

#### 3.2.2. Screening of Large Deletions/Duplications in the CDH1 Gene Using Multiplex Ligation-Dependent Probe Amplification (MLPA) Assay

**Figure 4:**
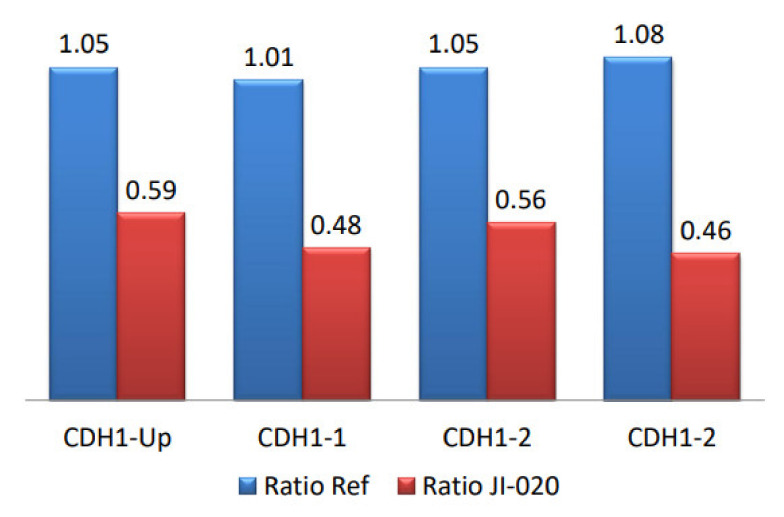
Detection of *CDH1* exon deletions by MLPA assay. (Blue) Control probes, (Red) Index cases harboring exon deletions (*CDH1* Exons 1 and 2).

Since heterozygous large deletions or duplications may remain undetected by conventional sequencing, we searched for possible rearrangements of the *CDH1* locus using the MLPA assay [30]. By comparing the control probes with the studied cases, we found that the DR of JI-020 was less than 0.7 for exons 1 and 2 showing abnormal MLPA features with more than a 45% reduction in signal, which indicates a gene dosage reduction. As shown in Figure 4 and Figure S1, JI-020 carried deletions at the 5′-end of the gene, spanning at least exons 1 and 2 from position 67325572 to 67329733. No other abnormalities were observed in the remaining patients.

#### 3.2.3. Molecular Modeling

**Figure 5:**
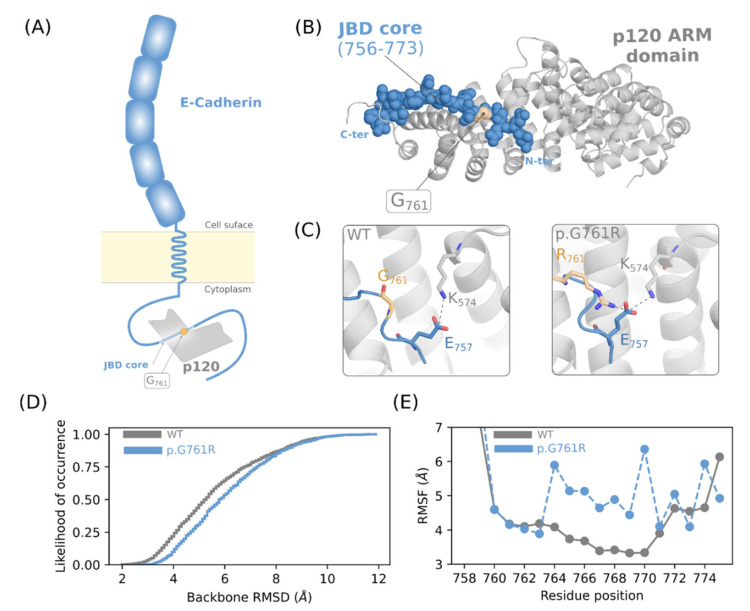
In silico analysis of p.G761R effect. (**A**) Schematic representation of the E-cadherin/p120 complex that includes the JMD core and the position of the mutation. (**B**) Co-crystal structure of the JMD core with p120 ARM domain showing the position of the mutated residue (light orange). (**C**) Interaction of G761 and R761 with the nearby amino acids in the WT form and the mutant form, respectively. (**D**) Cumulative likelihood of occurrence as a function of the backbone RMSD of the JMD core. All the structures of the ensembles were first fitted to the bound conformation of the JMD core prior to the calculation of the RMSD. (**E**) Root Mean Square Fluctuation (RMSF) profiles of the JMD core residues calculated for the WT and mutant forms.

The variant c.2281G > A occurs in the cytoplasmic tail of E-cadherin whose role is to regulate downstream cell–cell adhesion signaling (Figure 5A). The corresponding amino acid was solved as part of the juxta-membrane domain core region (JMD core) [31], which interacts with p120 catenin (p120) (Figure 5B). In the co-crystal structure, it corresponds to an 18-amino-acid peptide (residues 756–773) that interacts with the Armadillo (ARM) domain of p120. G761 interacts with the depth of the concavity formed by p120.

We first refined the JMD core_WT/ARM and JMD core_R761/ARM complexes to evaluate whether the mutation would significantly affect the peptide–protein interface. The complexes with the best ROSETTA scores showed a low Root Mean Square Deviation (RMSD) of 0.16 Angstroms. The refined wild-type model showed more favorable ROSETTA scores calculated from the 10 best conformations obtained with a median value of −640.55 and a standard deviation of 2.95. The mutant showed a less favorable median value of −574.784 and a standard deviation of 0.86. In addition, the in silico alanine scan analysis did not reveal that position 761 is a hotspot residue for interaction with the ARM domain (Table S5). However, we found that the R761 mutation-induced intrachain salt-bridge formation in the JMD core by pairing with E759, which partially interacts with K574. The latter paired only with K574 of the p120 ARM domain to form a salt bridge in the wild-type form (Figure 5C).

We then investigated the hypothesis that the conformational properties of the JMD core are affected by the mutation. We generated a trajectory of 1000 putative bound conformations for the WT and the mutant forms using MODPREP. We found that the WT structure was able to capture more conformations similar to the bound crystal shape after structural adjustment (Figure 5D). For example, seven conformations showed an RMSD value of less than 2.5 Angstroms, while the number increased to 30 Angstroms at a cutoff of 3 Angstroms. On the other hand, we reported zero and two conformations, respectively, for the same RMSD thresholds of the mutant form. From the ensemble, we calculated the Root Mean Square Fluctuation (RMSF) per amino acid of the JMD core (Figure 5E). We found that the WT form was more stable, while the mutant form showed an increase in flexibility for the R761 and G763-D868 segments. In addition, we found that R761 in the mutant form was able to form transient salt bridges with eight acidic residues of the JMD core, including D756, E757, E758, E762, E763, D764, D766, and D768, accounting for 5% of the total ensemble sampled. These residues represent the total acidic amino acids of the JMD core.

### 3.3. Molecular Analysis of CTNNA1 Gene

A total of 34 Tunisian patients with DGC were selected for screening for *CTNNA1* germline mutations. All identified variants are summarized in Table S6. All identified coding variants were synonymous, representing 8 out of 15. According to the ClinVar database, variants were classified as benign (53.33%), and a novel variant identified in two patients in intron 16 (c.2193-68C > T) was predicted to be a polymorphism.

### 3.4. Immunohistochemistry

The E-cadherin expression pattern was investigated by IHC in only 23/34 GC cases, for which the FFPE tumor tissues were available. Table 1 and Table S7 summarize the clinicopathologic features of the studied patients. Our results showed negative E-cadherin immunostaining in 30.43% (7/23) of cases versus 69.57% (16/23) of positive cases. The expression groups were classified as negative to weak expression (score 0–1), representing 39.13% (9/23) of cases with a normal membranous E-cadherin expression pattern in crypts and adjacent glandular cells (Figure 3B). The moderate expression group (score 2) included 21.74% (5/23) of cases, and the high expression group (score 3) included 39.13% (9/23) cases. The “abnormal” E-cadherin expression pattern includes both lost/reduced membranous expressions (Figure 3C,D).

## 4. Discussion

In the current study, we screened 34 DGC patients from unrelated families of the Northeast Tunisian region with suspected HDGC to shed light on the molecular basis of this disease. This region is known to have a relatively high proportion of digestive cancer syndromes and diffuse gastric tumors. The present research explores, for the first time, the mutational spectrum of *CDH1* and *CTNNA1* genes in Tunisian patients with HDGC fulfilling or not fulfilling IGCLC testing criteria. However, several researchers have found unexpected pathogenic and/or likely pathogenic *CDH1* germline variants (such as c.1003C.T (p.R335*) and c.1147C.T (p.Q383*)) in index cases that do not meet the 2015 IGCLC testing criteria [32,33,34]. Taking these findings into account, we selected 12 index cases not meeting the criteria in order to identify germline variants specific to our population.

To do so, we performed a screening of the coding region of both genes as well as *CDH1* large rearrangements. An IHC was used to investigate the E-cadherin protein expression profile in the available GC FFPE tissues as well. As a result, we identified a large pathogenic germline deletion and two likely pathogenic variants (a splice alteration and a missense variant) in the *CDH1* gene, as predicted by in silico analysis and molecular modeling. Approximately 10 to 20% of pathogenic variants are found in the *CDH1* gene for families meeting the IGCLC testing criteria [35,36], which is partially consistent with our results, as we found pathogenic and likely pathogenic *CDH1* variants in 13.64% of HDGC meeting clinical criteria patients. Compared with the literature, approximately 92% were already reported as described in Table S8.

**Figure 6:**
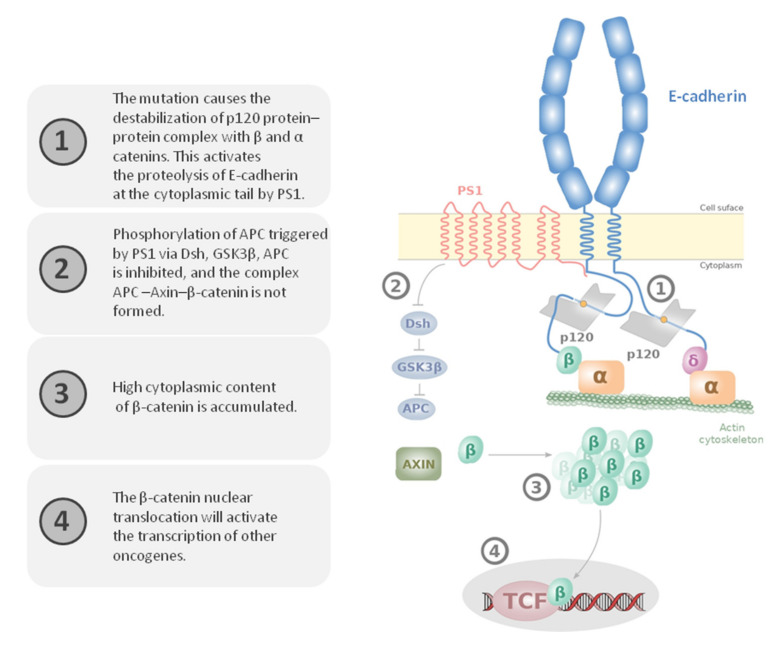
E-cadherin/β-catenin signaling pathway alteration in the presence of p.G761R (inspired from [43]).

The c.2281G > A variant is a very rare variant (ACMG-PM2). It has been previously reported [37], but this is the first time it was identified in a Tunisian patient. Structural bioinformatics analysis showed evidence in favor of a likely pathogenic effect for this variant. In fact, the c.2281G > A variant causes a shift in the conformational space of the E-cadherin protein. It allows a handful of conformations relevant to binding, while the free energy landscape is scanned, according to similar mechanisms described earlier [38,39]. This is consistent with glycine being endowed with more flexibility compared to arginine. This could allow a more efficient sampling of functionally relevant structures, including the bound form. Since glycine is able to form intrachain salt bridges with the acidic residues of the JMD core (Figure 5A,B), this variant could have a significant impact on the conformational space of the protein, thus also explaining the flexibility of the mutant form. Such a property would have a significant consequence by restricting the plasticity of the mutant form to conformations other than that of the WT form. Moreover, G761 has been shown to be highly conserved in the JMD core, and the GGG motif (residues 759–763) is crucial for the formation of a rotational structure that interacts with residues F437, W477, and N478 of p120 (ACMG-PP2-PP3) [31]. For JI-007 with the c.2281G > A *CDH1* variant, we observed a loss of E-cadherin protein expression by IHC in GC FFPE tissue. Indeed, the impairment of the protein–protein complex induced by the variant may explain reduced E-cadherin function, as predicted by in silico modeling analysis, which probably leads to HDGC. This is a major hallmark of tumor malignancy, which is induced by a variety of factors, including transcriptional regulation, mutation, and aberrant cadherin internalization (Figure 6) [40]. The ubiquitin-dependent endocytosis of E-cadherin [41] was associated with the depletion of E-cadherin from the cell surface [31], highlighted by the loss of membranous staining of E-cadherin in tumor cells in our results (Figure 3C,D).

The amino acid 761G is the third in a peptide sequence composed of 12 amino acids (from 758 to 769), which is crucial for the link of the E-cadherin cytoplasmic domain (ACMG-PM1) to PS1 and p120. This domain binds to β-catenin and inhibits the nuclear signaling pathway of this proto-oncogene. E-cadherin plays a pivotal role in the Wnt signal transduction pathway, causing the destabilization and the disassembly of the complexes E-cadherin, β-catenin, and p120 (Figure 6) [42].

Moreover, JI-007’s sDNA, examined by Sanger sequencing, showed a loss of heterozygosity for this variant. This is an additional criterion for classifying the variant as probably pathogenic (ACMG-PP4). Unfortunately, this variant was not tested in the index case’s relative to verify familial segregation, as they did not give their consent.

On the other hand, MLPA analysis showed that JI-020 displayed a large deletion from the 5′ locus, including exons 1 and 2 of the *CDH1* gene, implicating the signal peptide and part of the precursor domain of the E-cadherin protein. This large deletion is clearly pathogenic. Large *CDH1* deletions are rare and occur in only 4% of HDGC families [44]. A recent study reported that the 5′ breakpoint was 279 bp away from a breakpoint associated with the deletion of exons 1–2. Importantly, the immature molecule contains a short signal peptide and a precursor region preceding the extracellular domain prior to protein processing [45]. Signal peptides serve as docking sites for the signal recognition particle, the main molecule responsible for detecting the translocation code of secretory and membrane proteins [46,47,48]. The *CDH1* signal peptide core is essential for E-cadherin synthesis and delivery to extracytoplasmic regions. Failure in this checkpoint leads to the loss of protein expression and function and ultimately to disease [49]. Because of the unavailability of the tumor tissue, we were unable to perform an E-cadherin IHC to confirm this result. These findings highlight the critical importance of screening for large rearrangements of *CDH1* as well as *CDH1* variants for the management of HDGC families and individuals at high risk.

The index case JI-020 carried a large deletion of two exons of the *CDH1* gene. She has a sister diagnosed with breast cancer and a daughter diagnosed with colorectal cancer (Figure 1C and Table 2).

According to the literature, *CDH1* variants could have different clinical manifestations, as they may initiate different cancers. However, a recent study analyzed histology-specific associations between *CDH1* variants in DGC and LBC and found germline P/LP variants in the *CDH1* gene in 6.6% of patients with DGC and 0.3% of patients with LBC [50]. Several studies suggested that *CDH1* germline mutations are causative of a disease spectrum independent of the HDGC syndrome. It is a pleiotropic gene responsible for distinct clinical phenotypes: LBC, CRC, cleft lip/palate, and blepharocheilodontic syndrome [51,52,53].

The index case JI-014 carried the novel indel c.1565 + 3_1565 + 4delinsGT in intron 10, which is predicted to affect splicing. Indeed, the donor site was decreased 3 bps upstream with a percentage of −44.5% (MaxEnt: −64%; NN SPLICE: −25.1%, SSF: −16.8%). This indel alters the WT donor site, affects splicing, and activates an intronic cryptic donor site.

As recommended by IGCLC 2015 [14], identified *CDH1* variants should be submitted to the LOVD database in order to assess whether a given *CDH1* mutation has been found by others and whether it has been considered deleterious and likely disease-causative or not on the basis of population data, segregation analysis, in silico analysis and in vitro functional analysis, and/or recurrence in several individuals/families. For these reasons, we submitted all identified variants to the LOVD database.

The clinical utility of identifying the *CDH1* mutational spectrum determines whether unaffected relatives are at risk for developing DGC or LBC. Regarding carriers of the *CDH1* pathogenic variant, the updated recommendations are total prophylactic, reduced emphasis on prophylactic total gastrectomy for weak family history, and total gastrectomy for positive biopsies. If there is a family history of LBC, annual breast surveillance is recommended, and bilateral risk-reducing mastectomy with or without reconstruction should be considered [54].

In addition to *CDH1* variants, pathogenic variants in *CTNNA1* are known to occur in a small proportion of HDGC families. All identified coding variants in the current study were synonymous. Our results indicate that the genetic mutational profile of studied patients with suspected HDGC is different for families in other populations, as we did not find any reported *CTNNA1* mutations. These findings could be explained by the significant variability in GC frequency worldwide as well as risk factors [1]. Our findings highlight the particular genetic background of the Tunisian population compared to others [55,56,57,58,59].

## 5. Conclusions

The identification of hereditary cancer susceptibility genes is an essential step in understanding the basic molecular events of tumorigenesis and the clinical management of affected families. In this first Tunisian *CDH1* study, the frequency of identified variants was comparable to that reported in the literature with the presence of a novel large pathogenic deletion in the *CDH1* gene and a missense variant (c.2281G > A) having PM1, PM2, PP2, PP3, and PP4 criteria according to the ACMG classification. In light of these findings, we suggest reconsidering the ClinVar classification from VUS (Class3) to likely pathogenic (Class 4). Further functional studies or cosegregation analysis should be performed to confirm its pathogenicity.

## Figures and Tables

**Table 1:** Clinical pathological characteristics of 34 selected Patients.

	Total	
	N	%
Total	34	
Gender		
Male	13	38.24
Female	21	61.76
Age at diagnosis		
≤40	14	41.18
>40	20	58.82
Tumor subtype		
Diffuse	34	100
HP Status+		
Present	17	50
Absent	7	20.59
NI++	7	20.59
IGCLC 2015 Criteria *		
None	12	35.29
1	2	5.88
2	15	44.12
3	5	14.71
Stage		
NI	2	5.88
1	14	41.18
2	4	11.76
3	9	26.47
4	5	14.71

* (1) Two or more GC cases regardless of age, at least one confirmed DGC, in first-degree and second-degree relatives, (2) one case of DGC before 40 years old, (3) personal or familial history of DGC and LBC with at least one diagnosed before the age of 50 years. + HP: *Helicobacter Pylori*. ++ NI: Non-indicated.

**Table 2:** Characteristics of the predicted pathogenic and probably pathogenic variants.

*CDH1* Gene		Exons 1–2	Intron 10	Exon 14
Zoom in gene region		chr16: 67325572-67239733	c.1565 + 3_1565 + 4delinsGT	c.2281G > A
Method of identification		MLPA assay	Sanger Sequencing	Sanger Sequencing
Type of mutation		Deletion	Indel variant	Missense variant
Variant’s reference		Novel	Novel	rs779648243
ClinVar classification		NR	NR	VUS
Index case		JI-020	JI-014	JI-007
Clinicopathological characteristics of the patient	Age at diagnosis/sex	79/F	42/F	26/M
	TNM	T3N2M0	T4N1M1	T4N0M1
	Localization	NI	AP	F
	Personal history	DGC	DGC	DGC
	Familial history	CCR-BC	GC	No history
	HDGC criteria	3	2	2
E-cadherin expression		NA	Heterogeneous Loss	Homogenous Loss
Protein change		-	NA	p.G761R
Classification		D	PD	PD

F: Female; M: Male; VUS: Variant of Uncertain Significance; NR: Not Reported; NA: Not Applicable; PD: Probably Deleterious; D: Deleterious; BC: Breast Cancer; CCR: Colorectal Cancer; GC: Gastric Cancer; F: Fundic; AP: AntroPyloric.

## Data Availability

All data generated or analyzed during this study are included in this published article and its Additional file.

## Supplementary Materials

The following supporting information can be downloaded at: https://www.mdpi.com/article/10.3390/genes13030400/s1, Table S1: Settings of primer pairs of the *CDH1* gene. Table S2: Settings of primer pairs of the *CTNNA1* gene. Table S3: Detailed clinicopathological characteristics of 34 selected patients. Table S4: *CDH1* identified variants in DGC cases in the current study. Table S5: The in silico alanine scanning analysis; Table S6: *CTNNA1* identified variants in DGC cases in the current study; Table S7: E-cadherin expression status; Table S8: *CDH1* identified variants in DGC cases in the current study compared to other studies Refs [15,60,61,62,63,64,65,66,67,68,69,70,71,72,73,74,75,76,77,78] are cited in Table S8 File. Figure S1: Detection of *CDH1* exons deletions (Exons 1 and 2) by Coffalyser Software: A-B: Control probes, C-D: Index case JI-020 showing exons 1 and 2 heterozygous deletion.

## Abbreviations

HDGC	Hereditary Diffuse Gastric Carcinoma
HGC	Hereditary Gastric Carcinoma
MLPA	Multiplex Ligation Probe Amplification
IHC	Immunohistochemistry
GC/DGC	Gastric Carcinoma/Diffuse Gastric Carcinoma
FIGC	Familial Intestinal Gastric Cancer
DR	Dosage Ratio
BC/LBC	Breast Cancer/Lobular Breast Cancer
IGCLC	International Gastric Cancer Linkage Consortium
gDNA	Genomic DNA
sDNA	Somatic DNA
IRB	Institutional Review Board
ACMG	American College of Medical Genetics and Genomics
HP	*Helicobacter Pylori*
PDB	Protein Data Bank
SSF	Splice Site Finder-Like
ThSe	Sensitivity Threshold
ThSp	Specificity Threshold
FFPE	Formalin-Fixed Paraffin-Embedded
CCR	Colorectal Cancer
5′UTR	5′ Untranslated Region
B/LB	Benign/Likely Benign
VUS	Variant of Uncertain Significance
NR	Not Reported
NI	Not Indicated
ARM	Armadillo
JMD core	Juxta-Membrane Domain Core region
RMSF	Root Mean Square Fluctuation
RMSD	Root Mean Square Deviation
PM1/PM2	Moderate
PP2/PP3/PP4	Supporting

## References

[1] Bray F., Ferlay J., Soerjomataram I., Siegel R.L., Torre L.A., Jemal A. (2018). Global cancer statistics 2018: GLOBOCAN estimates of incidence and mortality worldwide for 36 cancers in 185 countries. CA Cancer J. Clin..

[2] Rawla P., Barsouk A. (2019). Epidemiology of gastric cancer: Global trends, risk factors and prevention. Gastroenterol. Rev..

[3] Van Cutsem E., Sagaert X., Topal B., Haustermans K., Prenen H. (2016). Gastric cancer. Lancet.

[4] Karimi P., Islami F., Anandasabapathy S., Freedman N.D., Kamangar F. (2014). Gastric Cancer: Descriptive Epidemiology, Risk Factors, Screening, and Prevention. Cancer Epidemiol. Biomark. Prev..

[5] Kelley J.R., Duggan J.M. (2003). Gastric cancer epidemiology and risk factors. J. Clin. Epidemiol..

[6] Asombang A.W. (2014). Gastric cancer in Africa: Current management and outcomes. World J. Gastroenterol..

[7] Crew K.D., Neugut A.I. (2006). Epidemiology of gastric cancer. World J. Gastroenterol..

[8] Lauren P. (1965). The Two Histological Main Types of Gastric Carcinoma: Diffuse and so-Called in-Testinal-Type Carcinoma. An Attempt at a Histo-Clinical Classification. Acta Pathol. Microbiol. Scand..

[9] Laurén P.A., Nevalainen T.J. (1993). Epidemiology of intestinal and diffuse types of gastric carcinoma a time-trend study in finland with comparison between studies from high-and low-risk areas. Cancer.

[10] Ma J., Shen H., Kapesa L., Zeng S. (2016). Lauren classification and individualized chemotherapy in gastric cancer. Oncol. Lett..

[11] Guilford P., Hopkins J., Harraway J., McLeod M., McLeod N., Harawira P., Taite H., Scoular R., Miller A.C., Reeve A.E. (1998). E-cadherin germline mutations in familial gastric cancer. Nature.

[12] Oliveira C., Pinheiro H., Figueiredo J., Seruca R., Carneiro F. (2015). Familial gastric cancer: Genetic susceptibility, pathology, and implications for management. Lancet Oncol..

[13] Park J.-G., Yang H.-K., Kim W.H., Caldas C., Yokota J., Guilford P.J. (2000). Report on the First Meeting of the International Collaborative Group on Hereditary Gastric Cancer. JNCI J. Natl. Cancer Inst..

[14] Van der Post R.S., Vogelaar I.P., Carneiro F., Guilford P., Huntsman D., Hoogerbrugge N., Caldas C., Schreiber K.E.C., Hardwick R.H., Ausems M.G.E.M. (2015). Hereditary diffuse gastric cancer: Updated clinical guidelines with an emphasis on germline CDH1 mutation carriers. J. Med. Genet..

[15] Hansford S., Kaurah P., Li-Chang H., Woo M., Senz J., Pinheiro H., Schrader K.A., Schaeffer D.F., Shumansky K., Zogopoulos G. (2015). Hereditary Diffuse Gastric Cancer Syndrome: CDH1 Mutations and Beyond. JAMA Oncol..

[16] Seevaratnam R., Coburn N., Cardoso R., Dixon M., Bocicariu A., Helyer L. (2011). A systematic review of the indications for genetic testing and prophylactic gastrectomy among patients with hereditary diffuse gastric cancer. Gastric Cancer.

[17] Stemmler M.P. (2008). Cadherins in development and cancer. Mol. Biosyst..

[18] Van Roy F., Berx G. (2008). The cell-cell adhesion molecule E-cadherin. Cell Mol. Life Sci..

[19] Majewski I., Kluijt I., Cats A., Scerri T.S., De Jong D., Kluin R.J.C., Hansford S., Hogervorst F.B.L., Bosma A.J., Hofland I. (2013). An α-E-catenin (CTNNA1) mutation in hereditary diffuse gastric cancer. J. Pathol..

[20] Lacroix L., Pautier P., Duvillard P., Motté N., Saulnier P., Bidart J.-M., Soria J.-C. (2006). Response of ovarian carcinomas to gefitinib-carboplatin-paclitaxel combination is not associated with EGFR kinase domain somatic mutations. Int. J. Cancer.

[21] Cordoni G., Woodward M.J., Wu H., Alanazi M., Wallis T., La Ragione R.M. (2016). Comparative genomics of European avian pathogenic *E. Coli* (APEC). BMC Genom..

[22] Den Dunnen J.T., Dalgleish R., Maglott D.R., Hart R.K., Greenblatt M.S., McGowan-Jordan J., Roux A.F., Smith T., Antonarakis S.E., Taschner P.E.M. (2016). HGVS Recommendations for the Description of Sequence Variants: 2016 Update. Hum. Mutat..

[23] Lee K., Krempely K., Roberts M.E., Anderson M.J., Carneiro F., Chao E., Dixon K., Figueiredo J., Ghosh R., Huntsman D. (2018). Specifications of the ACMG/AMP variant curation guidelines for the analysis of germline CDH1 sequence variants. Hum. Mutat..

[24] Richards S., Aziz N., Bale S., Bick D., Das S., Gastier-Foster J., Grody W.W., Hegde M., Lyon E., Spector E. (2015). Standards and guidelines for the interpretation of sequence variants: A joint consensus recommendation of the American College of Medical Genetics and Genomics and the Association for Molecular Pathology. Genet. Med..

[25] Leman R., Gaildrat P., Le Gac G., Ka C., Fichou Y., Audrezet M.-P., Caux-Moncoutier V., Caputo S.M., Boutry-Kryza N., Léone M. (2018). Novel diagnostic tool for prediction of variant spliceogenicity derived from a set of 395 combined in silico/in vitro studies: An international collaborative effort. Nucleic Acids Res..

[26] Šali A., Blundell T.L. (1993). Comparative Protein Modelling by Satisfaction of Spatial Restraints. J. Mol. Biol..

[27] Raveh B., London N., Schueler-Furman O. (2010). Sub-angstrom modeling of complexes between flexible peptides and globular proteins. Proteins Struct. Funct. Bioinform..

[28] Kortemme T., Kim D.E., Baker D. (2004). Computational Alanine Scanning of Protein-Protein Interfaces. Sci. STKE.

[29] McGibbon R.T., Beauchamp K.A., Harrigan M., Klein C., Swails J.M., Hernández C.X., Schwantes C.R., Wang L.-P., Lane T., Pande V.S. (2015). MDTraj: A Modern Open Library for the Analysis of Molecular Dynamics Trajectories. Biophys. J..

[30] Schouten J.P., McElgunn C.J., Waaijer R., Zwijnenburg D., Diepvens F., Pals G. (2002). Relative quantification of 40 nucleic acid sequences by multiplex ligation-dependent probe amplification. Nucleic Acids Res..

[31] Ishiyama N., Lee S.-H., Liu S., Li G.-Y., Smith M.J., Reichardt L.F., Ikura M. (2010). Dynamic and Static Interactions between p120 Catenin and E-Cadherin Regulate the Stability of Cell-Cell Adhesion. Cell.

[32] Katona B.W., Clark D.F., Domchek S.M. (2019). CDH1 on Multigene Panel Testing: Look Before You Leap. JNCI J. Natl. Cancer Inst..

[33] Huynh J.M., Laukaitis C.M. (2016). Panel testing reveals nonsense and missense CDH 1 mutations in families without hereditary diffuse gastric cancer. Mol. Genet. Genom. Med..

[34] Lowstuter K., Espenschied C.R., Sturgeon D., Ricker C., Karam R., LaDuca H., Culver J.O., Dolinsky J.S., Chao E., Sturgeon J. (2017). Unexpected CDH1 Mutations Identified on Multigene Panels Pose Clinical Management Challenges. JCO Precis. Oncol..

[35] Benusiglio P.R., Colas C., Rouleau E., Uhrhammer N., Romero P., Remenieras A., Moretta A., Wang Q., De Pauw A., Buecher B. (2015). Hereditary diffuse gastric cancer syn-drome: Improved performances of the 2015 testing criteria for the identification of probands with a CDH1 germline mutation. J. Med. Genet..

[36] Van der Post R.S., Vogelaar I.P., Manders P., van der Kolk L.E., Cats A., van Hest L.P., Sijmons R., Aalf C.M., Ausems M.G.E.M., Gómez García E.B. (2015). Accuracy of Hereditary Diffuse Gastric Cancer Testing Criteria and Outcomes in Patients with a Germline Mutation in CDH1. Gastroenterology.

[37] Momozawa Y., Iwasaki Y., Parsons M.T., Kamatani Y., Takahashi A., Tamura C., Katagiri T., Yoshida T., Nakamura S., Sugano K. (2018). Germline pathogenic variants of 11 breast cancer genes in 7051 Japanese patients and 11,241 controls. Nat. Commun..

[38] Grünberg R., Leckner J., Nilges M. (2004). Complementarity of Structure Ensembles in Protein-Protein Binding. Structure.

[39] Paul F., Weikl T.R. (2016). How to Distinguish Conformational Selection and Induced Fit Based on Chemical Relaxation Rates. PLoS Comput. Biol..

[40] Mosesson Y., Mills G.B., Yarden Y. (2008). Derailed endocytosis: An emerging feature of cancer. Nat. Cancer.

[41] Fujita Y., Krause G., Scheffner M., Zechner D., Leddy H.E.M., Behrens J., Sommer T., Birchmeier W. (2002). Hakai, a c-Cbl-like protein, ubiquitinates and induces endocytosis of the E-cadherin complex. Nat. Cell Biol..

[42] Marambaud P., Shioi J., Serban G., Georgakopoulos A., Sarner S., Nagy V., Baki L., Wen P., Efthimiopoulos S., Shao Z. (2002). A presenilin-1/gamma-secretase cleavage releases the E-cadherin intracellular domain and regulates disassembly of adherens junctions. EMBO J..

[43] Tian X., Liu Z., Niu B., Zhang J., Tan T.K., Lee S.R., Zhao Y., Harris D.C.H., Zheng G. (2011). E-Cadherin/β-Catenin Complex and the Epithelial Barrier. J. Biomed. Biotechnol..

[44] Oliveira C., Senz J., Kaurah P., Pinheiro H., Sanges R., Haegert A., Corso G., Schouten J., Fitzgerald R., Vogelsang H. (2009). Germline CDH1 deletions in hereditary diffuse gastric cancer families. Hum. Mol. Genet..

[45] Paredes J., Figueiredo J., Albergaria A., Oliveira P., Carvalho J., Ribeiro A.S., Caldeira J., Costa A., Correia J.S., Oliveira M.J. (2012). Epithelial E- and P-cadherins: Role and clinical significance in cancer. Biochim. Biophys. Acta.

[46] Ehsan A., Mahmood K., Khan Y.D., Khan S.A., Chou K.-C. (2018). A Novel Modeling in Mathematical Biology for Classification of Signal Peptides. Sci. Rep..

[47] Halic M., Becker T., Pool M., Spahn C.M.T., Grassucci R.A., Frank J., Beckmann R. (2004). Structure of the signal recognition particle interacting with the elongation-arrested ribosome. Nature.

[48] Janda C.Y., Li J., Oubridge C., Hernández H., Robinson C., Nagai K. (2010). Recognition of a signal peptide by the signal recognition particle. Nature.

[49] Figueiredo J., Melo S., Gamet K., Godwin T., Seixas S., Sanches J.M., Guilford P., Seruca R. (2018). E-cadherin signal sequence disruption: A novel mechanism underlying hereditary cancer. Mol. Cancer.

[50] Adib E., El Zarif T., Nassar A.H., Akl E.W., Alaiwi S.A., Mouhieddine T.H., Esplin E.D., Hatchell K., Nielsen S.M., Rana H.Q. (2021). CDH1 germline variants are enriched in patients with colorectal cancer, gastric cancer, and breast cancer. Br. J. Cancer.

[51] Figueiredo J., Melo S., Carneiro P., Moreira A.M., Fernandes S., Ribeiro A.S., Guilford P., Paredes J., Seruca R. (2019). Clinical spectrum and pleiotropic nature of CDH1 germline mutations. J. Med. Genet..

[52] Vogelaar I.P., Figueiredo J., van Rooij I.A.L.M., Simões-Correia J., van der Post R.S., Melo S., Seruca R., Carels C.E., Ligtenberg M.J., Hoogerbrugge N. (2013). Identification of germline mutations in the cancer predisposing gene CDH1 in patients with orofacial clefts. Hum. Mol. Genet..

[53] Xie Z.M., Li L.S., Laquet C., Xie X.M., Penault-Llorca F., Uhrhammer N., Bignon Y.J. (2011). Germline mutations of the E-cadherin gene in families with inherited invasive lobular breast carcinoma but no diffuse gastric cancer. Cancer.

[54] Blair V.R., McLeod M., Carneiro F., Coit D.G., D’Addario J.L., van Dieren J.M., Harris K.L., Hoogerbrugge N., Oliveira C., van der Post R.S. (2020). Hereditary diffuse gastric cancer: Updated clinical practice guidelines. Lancet Oncol..

[55] Boujemaa M., Hamdi Y., Mejri N., Romdhane L., Ghedira K., Bouaziz H., Benna H.E., Labidi S., Dallali H., Jaidane O. (2021). Germline copy number variations in BRCA1/2 negative families: Role in the molecular etiology of hereditary breast cancer in Tunisia. PLoS ONE.

[56] Hamdi Y., Boujemaa M., Ben Rekaya M., Ben Hamda C., Mighri N., El Benna H., Mejri N., Labidi S., Daoud N., The PEC Consortium (2018). Family specific genetic predisposition to breast cancer: Results from Tunisian whole exome sequenced breast cancer cases. J. Transl. Med..

[57] Jaballah-Gabteni A., Tounsi H., Kabbage M., Hamdi Y., Elouej S., Ben Ayed I., Medhioub M., Mahmoudi M., Dallali H., Yaiche H. (2019). Identification of novel pathogenic MSH2 mutation and new DNA repair genes variants: Investigation of a Tunisian Lynch syndrome family with discordant twins. J. Transl. Med..

[58] Mighri N., Hamdi Y., Boujemaa M., Othman H., Ben Nasr S., El Benna H., Mejri N., Labidi S., Ayari J., Jaidene O. (2020). Identification of Novel BRCA1 and RAD50 Mutations Associated with Breast Cancer Predisposition in Tunisian Patients. Front. Genet..

[59] Mezzi N., Messaoud O., Mkaouar R., Zitouna N., Romdhane S., Abdessalem G., Charfeddine C., Maazoul F., Ouerteni I., Hamdi Y. (2021). Spectrum of Genetic Diseases in Tunisia: Current Situation and Main Milestones Achieved. Genes.

[60] Avizienyte E., Launonen V., Salovaara R., Kiviluoto T., Aaltonen L.A. (2001). E-cadherinis not frequently mutated in hereditary gastric cancer. J. Med. Genet..

[61] Bacani J.T., Soares M., Zwingerman R., Di Nicola N., Senz J., Riddell R., Huntsman D.G., Gallinger S. (2006). CDH1/E-cadherin germline mutations in early-onset gastric cancer. J. Med. Genet..

[62] Mucaki E.J., Caminsky N.G., Perri A.M., Lu R., Laederach A., Halvorsen M., Knoll J.H.M., Rogan P.K. (2016). A unified analytic framework for prioritization of non-coding variants of uncertain significance in heritable breast and ovarian cancer. BMC Med Genomics..

[63] El-Husny A., Raiol-Moraes M., Amador M., Ribeiro-dos-Santos A.M., Montagnini A., Barbosa S., Silva A., Assumpção P., Ishak G., Santos S. (2016). CDH1 mutations in gastric cancer patients from northern Brazil identified by Next- Generation Sequencing (NGS). Genet. Mol. Biol..

[64] Guindalini R.S.C., Cormedi M.C.V., Maistro S., Pasini F.S., Branas P.C.A.A., dos Santos L., Pereira G.F.d., de Bock G.H., Saccaro D.M., Katayama M.L.H. (2019). Frequency of CDH1 germline variants and contribution of dietary habits in early age onset gastric cancer patients in Brazil. Gastric Cancer.

[65] Norero E., Alarcon M.A., Hakkaart C., de Mayo T., Mellado C., Garrido M., Aguayo G., Lagos M., Torres J., Calvo A. (2019). Identification of c.1531C>T Pathogenic Variant in the *CDH1* Gene as a Novel Germline Mutation of Hereditary Diffuse Gastric Cancer. Int. J. Mol. Sci..

[66] Simoes-Correia J., Figueiredo J., Oliveira C., van Hengel J., Seruca R., Van Roy F., Suriano G. (2008). Endoplasmic reticulum quality control: a new mechanism of E-cadherin regulation and its implication in cancer. Hum. Mol. Genet..

[67] Brooks-Wilson A.R. (2004). Germline E-cadherin mutations in hereditary diffuse gastric cancer: assessment of 42 new families and review of genetic screening criteria. J. Med. Genet..

[68] Johnston J.J., Rubinstein W.S., Facio F.M., Ng D., Singh L.N., Teer J.K., Mullikin J.C., Biesecker L.G. (2012). Secondary variants in individuals undergoing exome sequencing: screening of 572 individuals identifies high-penetrance mutations in cancer-susceptibility genes. Am. J. Hum. Genet..

[69] Suriano G., Seixas S., Rocha J., Seruca R. (2006). A model to infer the pathogenic significance of CDH1 germline missense variants. J. Mol. Med. Berl. Ger..

[70] Bustos-Carpinteyro A.R., Oliveira C., Sousa A., Oliveira P., Pinheiro H., Carvalho J., Magaña-Torres M.T., Flores-Miramontes M.G., Aguilar-Lemarroy A., Jave-Suárez L.F. (2019). CDH1 somatic alterations in Mexican patients with diffuse and mixed sporadic gastric cancer. BMC Cancer.

[71] Oliveira C., Bordin M.C., Grehan N., Huntsman D., Suriano G., Machado J.C., Kiviluoto T., Aaltonen L., Jackson C.E., Seruca R. (2002). Screening E-cadherin in gastric cancer families reveals germline mutations only in hereditary diffuse gastric cancer kindred. Hum. Mutat..

[72] Jalkh N., Chouery E., Haidar Z., Khater C., Atallah D., Ali H., Al-Mulla M.R., Al-Mulla F., Megarbane A. (2017). Next-generation sequencing in familial breast cancer patients from Lebanon. BMC Med. Genomics..

[73] Keller G., Vogelsang H., Becker I., Plaschke S., Ott K., Suriano G., Mateus A.R., Seruca R., Biedermann K., Huntsman D. (2004). Germline mutations of the E-cadherin(CDH1) and TP53 genes, rather than of RUNX3 and HPP1, contribute to genetic predisposition in German gastric cancer patients. J. Med Genet..

[74] Salahshor S., Haixin L., Huo H., Kristensen V.N., Loman N., Sjöberg-Margolin S., Borg Å., Børresen-Dale An., Vorechovsky I., Lindblom A. (2001). Low frequency of E-cadherin alterations in familial breast cancer. Breast Cancer Res. BCR..

[75] Humar B., Toro T., Graziano F., Müller H., Dobbie Z., Kwang-Yang H., Eng C., Hampel H., Gilbert D., Winship I. (2002). Novel germlineCDH1mutations in hereditary diffuse gastric cancer families. Hum. Mutat..

[76] Kheirollahi M., Saneipour M., Tabatabaiefar M.A., Zeinalian M., Minakari M., Moridnia A. (2020). New Variants in the CDH1 Gene in Iranian Families with Hereditary Diffuse Gastric Cancer. Middle East J. Cancer.

[77] Moridnia A., Tabatabaiefar M.A., Zeinalian M., Minakari M., Kheirollahi M., Moghaddam N.A. (2018). Novel Variants and Copy Number Variation in CDH1 Gene in Iranian Patients with Sporadic Diffuse Gastric Cancer. J. Gastrointest. Cancer.

[78] Barber M., Murrell A., Ito Y., Maia A.-T., Hyland S., Oliveira C., Save V., Carneiro F., Paterson A.L., Grehan N. (2008). Mechanisms and sequelae of E-cadherin silencing in hereditary diffuse gastric cancer. J. Pathol..

