# Supplementary material for: CDH1 Germline Variants in a Tunisian Cohort with Hereditary Diffuse Gastric Carcinoma
Source: Genes (Basel). 2022 Feb 23;13(3):400. doi: 10.3390/genes13030400 (PMC8950196; doi:10.3390/genes13030400)
Supplement: Supplementary file 1 [file genes-13-00400-s001.zip › Figure S1.pdf]

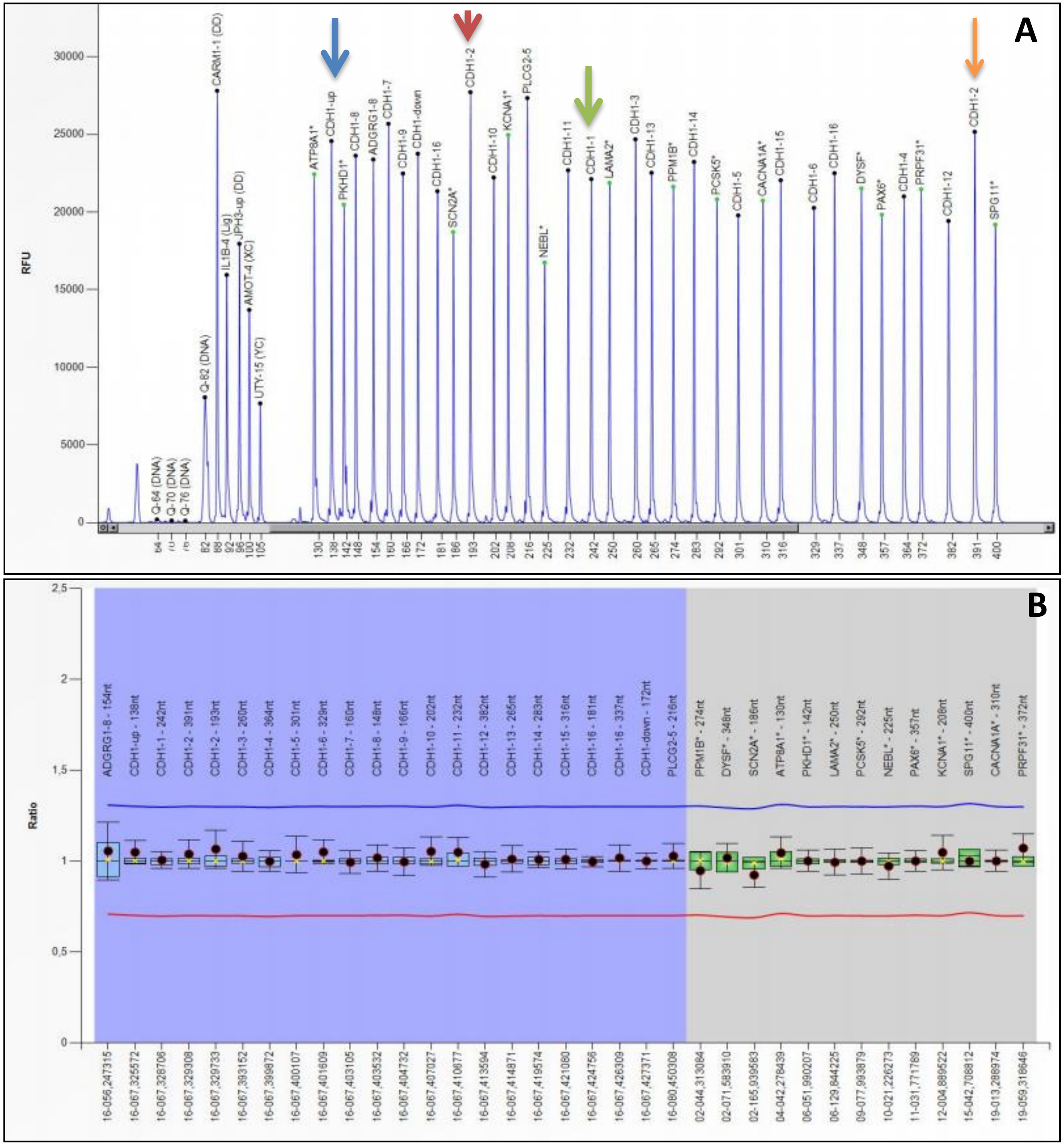

- Figure S1. (A-B) Control probes. The spots represent MLPA probes.
- The lower red line indicates a peak ratio of 0.75 and any probes (red spot) below this line represent deletions.

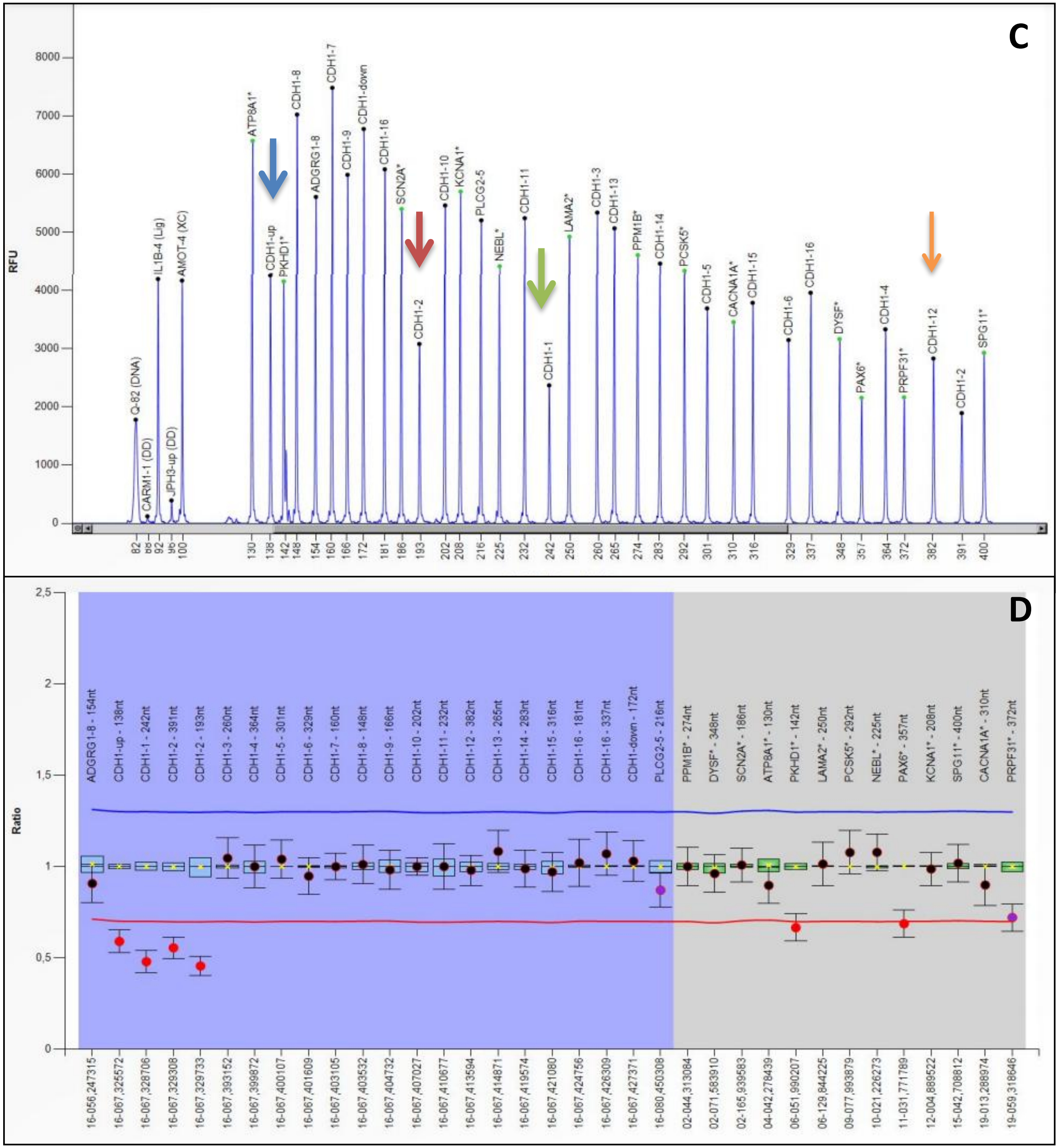

- Figure S1. (C-D) Index case "JI-020" having Exons 1 and 2 deletions (represented by different arrows).
- The lower red line indicates a peak ratio of 0.75 and any probes (red spot) below this line represent deletions.
