# Supplementary material for: CDH1 Germline Variants in a Tunisian Cohort with Hereditary Diffuse Gastric Carcinoma
Source: Genes (Basel). 2022 Feb 23;13(3):400. doi: 10.3390/genes13030400 (PMC8950196; doi:10.3390/genes13030400)
Supplement: Supplementary file 1 [file genes-13-00400-s001.zip › Table S8.pdf]

CDH1 variants identified in DGC cases in the current study compared to other studies.

N	Mutation	Ethnicity or Country	N of GC Cases	Age of diagnosis	N of BC Cases	References
**1**	c.-71C>G	Finnish	One/13 (7.7%)	NI	NI	[1]
		Canadian	one/58 (3.4%)	NI	NI	[2]
		Canadian	Two/51 (3.9%)	NI	NI	[3]
**2**	c.48+6 C>T	Brazil	Four/Six	NI	NI	[4]
		Brazil	78/88	NI	NI	[5]
		Chile	36/36	NI	NI	[6]
**3**	c.345G>A	Brazil	One/88	NI	NI	[5]
**4**	c.387+27C>T	Brazil	One/88	NI	NI	[5]
**5**	c.531+10G>C	Brazil	Four/88	NI	NI	[5]
		Chile	1/36	NI	NI	[6]
**6**	c.532-18C>T	Brazil	NI	NI	NI	[5]
		Portugal	NI	NI	NI	[7]
**7**	c.892G>A	European	3	36/32/33	0	[8]
		Eurpean	NI	NI	NI	[9]
		Polish	1	61	0	[10]
		Caucasian	NI	NI	NI	[11]
**8**	c.1320+45G>C	NF	-	-	-	NF
**9**	c.1566-88C>T	NF	-	-	-	NF
**10**	c.1566-80C>G	NF	-	-	-	NF
**11**	c.1680 G>C	Mexican	5%	NA	NA	[12]
**12**	c.1712-52G>C	NF	-	-	-	NF

**13**	c.1896C>T	Brazil	7/88	NI	NI	[5]
		NI	GC : One/34 (2.9%) / CC : 5/100 (5%)	NI	NI	[13]
**14**	c.1774G>A	Lebanese	NI	NI	One	[14]
		German	NI	NI	NI	[15]
		Canadian	0.12	NI	NI	[3]
		Sweedan	0.1 CCR	70	0	[16]
**15**	c.1937-13T>C	Brazil	13/88	NI ;NI	NI.NI	[5]
		NI	GC : Two/27 (7.4%) / CC : 25/100 (25%)	NI	NI	[13]
**16**	c.2076T>C	Finnish	8/13 (61,5%)	NI	NI	[1]
		Brazil	6/6 (100%)	NI	NI	[4]
		Brazil	78/88	NI	NI	[5]
		Italian	GC : 22/46 (47.8%) / CC : 34/69 (49.3%)	NI	NI	[17]
		Chile	30/36	NI	NI	[6]
		NI	GC : 15/27 (55.6%) / CC : 29/100 (59%)	NI	NI	[13]
**17**	c.2281 G>A	NF	-	-	-	NF
**18**	c.2295+166C>T	NF	-	-	-	NF
**19**	c.2439+52 G>A	Iran	NI	NI	NI	[18]
		Iran	4/28	NI	NI	[19]

**20**	c.2520C>T					[20]
**21**	c.2634C>T	Northern Brazil	One/Six	NI	NI	[4]
		Brazil	Four/88 :	NI	NI	[5]
		NI	1/34 (2.9%)	NI	NI	[13]

NF : Not Found ; NI : Non Indicated ; GC : Gastric Cancer Cases ; CC : Control Cases ; BC : Breast Cancer Cases

References:

1. Avizienyte E, Launonen V, Salovaara R, Kiviluoto T, Aaltonen LA. E-cadherinis not frequently mutated in hereditary gastric cancer. J Med Genet. 2001;38:49–52.

2. Bacani JT, Soares M, Zwingerman R, Di Nicola N, Senz J, Riddell R, et al. CDH1/E-cadherin germline mutations in early-onset gastric cancer. J Med Genet. 2006;43:867–72.

3. Mucaki EJ, Caminsky NG, Perri AM, Lu R, Laederach A, Halvorsen M, et al. A unified analytic framework for prioritization of non-coding variants of uncertain significance in heritable breast and ovarian cancer. BMC Med Genomics. 2016;9:19.

4. El-Husny A, Raiol-Moraes M, Amador M, Ribeiro-dos-Santos AM, Montagnini A, Barbosa S, et al. CDH1 mutations in gastric cancer patients from northern Brazil identified by Next- Generation Sequencing (NGS). Genet Mol Biol. 2016;39:189–98.

5. Guindalini RSC, Cormedi MCV, Maistro S, Pasini FS, Branas PCAA, dos Santos L, et al. Frequency of CDH1 germline variants and contribution of dietary habits in early age onset gastric cancer patients in Brazil. Gastric Cancer. 2019;22:920–31.

6. Norero E, Alarcon MA, Hakkaart C, de Mayo T, Mellado C, Garrido M, et al. Identification of c.1531C>T Pathogenic Variant in the CDH1 Gene as a Novel Germline Mutation of Hereditary Diffuse Gastric Cancer. Int J Mol Sci. 2019;20:4980.

7. Simoes-Correia J, Figueiredo J, Oliveira C, van Hengel J, Seruca R, Van Roy F, et al. Endoplasmic reticulum quality control: a new mechanism of E-cadherin regulation and its implication in cancer. Hum Mol Genet. 2008;17:3566–76.

8. Brooks-Wilson AR. Germline E-cadherin mutations in hereditary diffuse gastric cancer: assessment of 42 new families and review of genetic screening criteria. J Med Genet. 2004;41:508–17.

9. Hansford S, Kaurah P, Li-Chang H, Woo M, Senz J, Pinheiro H, et al. Hereditary Diffuse Gastric Cancer Syndrome: CDH1 Mutations and Beyond. JAMA Oncol. 2015;1:23–32.

10. Johnston JJ, Rubinstein WS, Facio FM, Ng D, Singh LN, Teer JK, et al. Secondary variants in individuals undergoing exome sequencing: screening of 572 individuals identifies high-penetrance mutations in cancer-susceptibility genes. Am J Hum Genet. 2012;91:97–108.

11. Suriano G, Seixas S, Rocha J, Seruca R. A model to infer the pathogenic significance of CDH1 germline missense variants. J Mol Med Berl Ger. 2006;84:1023–31.

12. Bustos-Carpinteyro AR, Oliveira C, Sousa A, Oliveira P, Pinheiro H, Carvalho J, et al. CDH1 somatic alterations in Mexican patients with diffuse and mixed sporadic gastric cancer. BMC Cancer. 2019;19:69.

13. Oliveira C, Bordin MC, Grehan N, Huntsman D, Suriano G, Machado JC, et al. Screening E-cadherin in gastric cancer families reveals germline mutations only in hereditary diffuse gastric cancer kindred. Hum Mutat. 2002;19:510–7.

14. Jalkh N, Chouery E, Haidar Z, Khater C, Atallah D, Ali H, et al. Next-generation sequencing in familial breast cancer patients from Lebanon. BMC Med Genomics. 2017;10:8.

15. Keller G, Vogelsang H, Becker I, Plaschke S, Ott K, Suriano G, et al. Germline mutations of the E-cadherin (CDH1) and TP53 genes, rather than of RUNX3 and HPP1, contribute to genetic predisposition in German gastric cancer patients. J Med Genet. 2004;41:e89–e89.

16. Salahshor S, Haixin L, Huo H, Kristensen VN, Loman N, Sjöberg-Margolin S, et al. Low frequency of E-cadherin alterations in familial breast cancer. Breast Cancer Res BCR. 2001;3:199–207.

17. Humar B, Toro T, Graziano F, Müller H, Dobbie Z, Kwang-Yang H, et al. Novel germline CDH1 mutations in hereditary diffuse gastric cancer families. Hum Mutat. 2002;19:518–25.

18. Kheirollahi M, Saneipour M, Tabatabaiefar MA, Zeinalian M, Minakari M, Moridnia A. New Variants in the CDH1 Gene in Iranian Families with Hereditary Diffuse Gastric Cancer. Middle East J Cancer. 2020;11:493–501.

19. Moridnia A, Tabatabaiefar MA, Zeinalian M, Minakari M, Kheirollahi M, Moghaddam NA. Novel Variants and Copy Number Variation in CDH1 Gene in Iranian Patients with Sporadic Diffuse Gastric Cancer. J Gastrointest Cancer. 2018. https://doi.org/10.1007/s12029-018-0082-7.

20. Barber M, Murrell A, Ito Y, Maia A-T, Hyland S, Oliveira C, et al. Mechanisms and sequelae of E-cadherin silencing in hereditary diffuse gastric cancer. J Pathol. 2008;216:295–306.
